# Ligustrazine Exerts Cardioprotection in Animal Models of Myocardial Ischemia/Reperfusion Injury: Preclinical Evidence and Possible Mechanisms

**DOI:** 10.3389/fphar.2018.00729

**Published:** 2018-07-25

**Authors:** Qun Zheng, Yue-yue Huang, Peng-chong Zhu, Qiang Tong, Xiao-yi Bao, Qi-hao Zhang, Guo-qing Zheng, Yan Wang

**Affiliations:** Department of Internal Medicine, The Second Affiliated Hospital and Yuying Children's Hospital of Wenzhou Medical University, Wenzhou, China

**Keywords:** ligustrazine, myocardial ischemia/reperfusion injury, efficacy, mechanisms, meta-analysis

## Abstract

Ligustrazine (Lig) is one of the main effective components of Ligusticum Chuanxiong Hort, which possesses a variety of biological activities in the cardiovascular system. Here, we conducted a preclinical systematic review to investigate the efficacy of Lig for animal models of myocardial ischemia/reperfusion injury and its possible mechanisms. Twenty-five studies involving 556 animals were identified by searching 6 databases from inception to August 2017. The methodological quality was assessed by using Collaborative Approach to Meta-Analysis and Review of Animal Data from Experimental Studies (CAMARADES) 10-item checklist. All the data were analyzed using Rev-Man 5.3 software. As a result, the score of study quality ranged from 2 to 6 points. Meta-analyses showed Lig can significantly decrease the myocardial infarct size, cardiac enzymes and troponin compared with control (*P* < 0.01). The possible mechanisms of Lig for myocardial infarction are antioxidant, anti-inflammatory, anti-apoptosis activities and improving coronary blood flow and myocardial metabolism. In conclusion, the findings indicated that Lig exerts cardio protection through multiple signaling pathways in myocardial ischemia/reperfusion injury.

## Introduction

Myocardial infarction (MI) is one of the most common causes of death and disability worldwide (Mozaffarian et al., 2015). The injuries inflicted on the myocardium during acute MI are the result of two processes: ischemia and subsequent reperfusion [ischemia/reperfusion (I/R) injury] (Ibáñez et al., 2015). In patients with MI, the treatment of choice for reducing acute myocardial ischemic injury and limiting MI size is timely and effective myocardial reperfusion using either thombolytic therapy or primary percutaneous coronary intervention (PPCI) (Hausenloy and Yellon, 2013). However, abrupt restoration therapy of coronary flow can lead to possible result in adverse events (Heusch and Gersh, 2017) such as reversible impairment of myocardial contractility (myocardial stunning), ventricular arrhythmias, and microvascular dysfunction, for which there is still no effective therapy (Hausenloy and Yellon, 2013). Several strategies have been developed to attenuate and/or modulate the extent of the I/R injury associated with cardiopulmonary attack for years (Chun et al., 2011); however, results of clinical trials disappoint us, and there are some large clinical trials evaluating promising interventions from bench to bedside that have just begun (Frank et al., 2012; Schmidt et al., 2015). Thus, it is urgent to seek new cardioprotective strategies to improve myocardial salvage and cardiac function when myocardial I/R injury happen.

**Figure 1:**
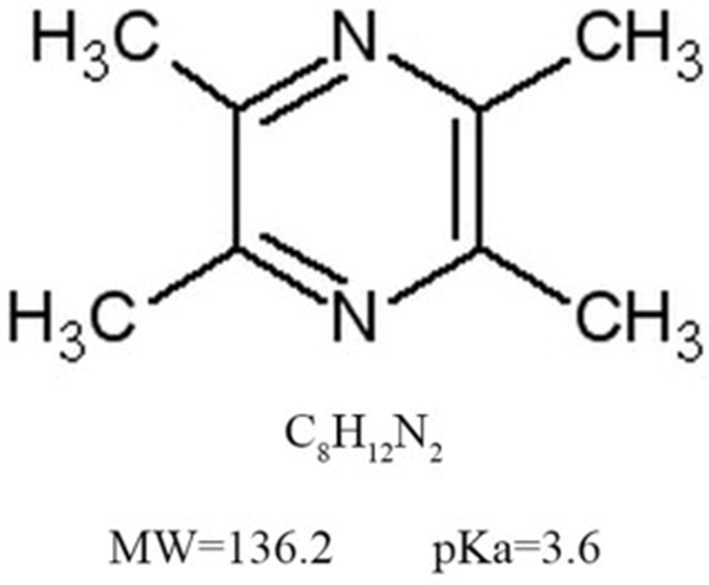
The chemical structure of ligustrazine. The molecular formula of ligustrazineis C8H12N2. The molecular weight of ligustrazine is 136.2, and the value of pKa is 3.6.

Chuanxiong, Rhizoma Ligustici Chuanxiong, sichuan lovage rhizome, the dried rhizomes of Ligusticum chuanxiong Hort., a perennial herbal plant of the *Umbelliferae/Apiaceae*family, has the function of activating blood and promoting Qi, first recorded in the earliest complete Pharmacopoeia of China, *Shennong Bencao Jing* (*Shennong's Classic of Materia Medica*) from Warring States Period to Han Dynasty. Qi, an important concept in *Huangdi Neijing* (*Huangdi's Internal Classic*) written in AD 206~221, is of the vital substances to comprise body and is the vital energy to maintain life (vital Qi), whereas the exogenous pathogenic factors and/or endogenous pathological changes in the body leads to varieties of diseases (pathogenic Qi) (Chen and Chen, 1998; Yuan et al., 2013). Thus, to promote Qi is a key treatment method in traditional Chinese medicine. Chuanxiong has been widely used in the treatment of cardiovacular diseases for thousands of years and is still widely used in modern time due to its extensive biological activities (Lu et al., 2015). Ligustrazine (Lig) (Figure 1) is one of the main effective components of Chuanxiong, which exerts potential cardio/cerebrovascular protective effects (Zhang et al., 2014; Xu et al., 2017). Lig have indicated that Lig and its numerous metabolites have outstanding pharmacokinetic characteristics, such as rapid metabolism, broad distribution and no accumulated toxic effect (Zou et al., 2018). In addition, animal models are invaluable tools for enriching our understanding of the mechanisms, etiology and treatment of human diseases (Sena et al., 2014). Preclinical systematic review can evaluate the efficacy of drugs more systematically, establish a test field for further animal experiments, provide reliable information for drug research, and lay a foundation for future clinical research (Sena et al., 2014; Disma et al., 2016; Zhang et al., 2017). Thus, we aim to evaluate the available preclinical evidence and possible mechanisms of Lig on cardioprotection in animal experiments of myocardial I/R injury.

## Methods

### Data sources and search strategy

Preferred Reporting Items for Systematic Review and Meta-Analyses (PRISMA) statement was abided (Stewart et al., 2015). We electronically searched in PubMed, EMBASE, Science Direct, Web of Science, Wanfang data Information Site, Chinese National Knowledge Infrastructure (CNKI), and VIP information database from inception to the end of August, 2017 using the following key words: “ligustrazine (MeSH Terms) OR tetramethylpyrazine (MeSH Terms) OR Ligustrazine (Title/Abstract) OR tetramethylpyrazine (Title/Abstract)” AND “myocardial infarction OR myocardial ischemia OR myocardial ischemia/reperfusion injury OR myocardial I/R injury.” Additional studies were identified through the reference lists of relevant reports. All the studies included were limited on animals.

### Study selection and data extraction

Two investigators (Zheng Q and Zhu PC) independently screened the titles and/or abstracts, of the search results and assessed the remaining full-text articles for eligibility. Any uncertainty eligibility was resolved by discussion. Studies were eligible for our systematic review if they met: (1) Lig for animal models of myocardial I/R injury established by ligating of the left anterior descending coronary artery (LAD) or injecting intravenously vasoconstrictor; (2) Analyzed interventions was received Lig as monotherapy at any dose. Comparator interventions were isasteric and non-functional liquid (normal saline) or no treatment; (3) the primary outcome measures were MI size and/or cardiac enzymes and/or cardiac troponin T (cTnT) and/or the level of ST-segment depression and/or left ventricular ejection fraction (LVEF) and/or shortening fraction (FS). The secondary outcome measures were mechanisms of Lig for myocardial I/R injury. Prespecified exclusion criteria were the treatment of Lig in conjunction with other compounds or Lig-based prescriptions, non-myocardial ischemia model, no control group, not published in peer-review journals, and duplicate publications. In the case of multiple publications from one study, we chose the articles with the largest sample or the earliest publication.

Two independent investigators (Zheng Q and Huang YY) extracted the following details from the included studies: (1) name of first author and year of publication; (2) details (species, number, sex, and weight) of animals for each study; (3) methods to establish animal models of myocardial I/R, and the anesthesia methods for model preparation; (4) the information of treatment group, including therapeutic drug dosage, method of administration, duration of treatment, and the same information of control group; (5) mean value and standard deviation of outcomes. The data of highest dose was selected when the treatment group included various doses of the target drug. The result of the peak time point was included when the data were expressed at different times.

### Risk of bias in individual studies

The methodological quality of each included study was evaluated by using Collaborative Approach to Meta-Analysis and Review of Animal Data from Experimental Studies (CAMARADES) 10-item checklist (Macleod et al., 2004) with minor modification (Yu et al., 2017) as follows: A: peer-reviewed publication; B: control of temperature; C: random allocation to treatment or control; D: blinded induction of model; E: blinded assessment of outcome; F: use of anesthetic without significant intrinsic cardioprotective activity; G: appropriate animal model (aged, diabetic, or hypertensive); H: sample size calculation; I: compliance with animal welfare regulations; and J: statement of potential conflict of interests. Every item was given one point. Two investigators (P.C. Z and Y.Y. H) independently evaluated the study quality and divergences were well settled through consulting with correspondence authors.

### Statistical analysis

Meta-analyses and sub-analyses were performed using RevMan V.5.3 software. Outcome measures were all considered as continuous data and given an estimate of the combined overall effect sizes utilizing standard mean difference (SMD) or mean difference (MD) with the effects model. SMD or MD with its 95% confidence interval (CI) was used to assess the strength of efficacy of Lig for myocardial I/R injury. The *I*^2^ statistic was used for assessment of heterogeneity among individual studies. A fixed effects model (*I*^2^ < 50%) or a random effects model (*I*^2^ > 50%) was used depending on the value of *I*^2^. Probability value *p* < 0.05 was considered significant.

## Results

### Study inclusion

**Figure 2:**
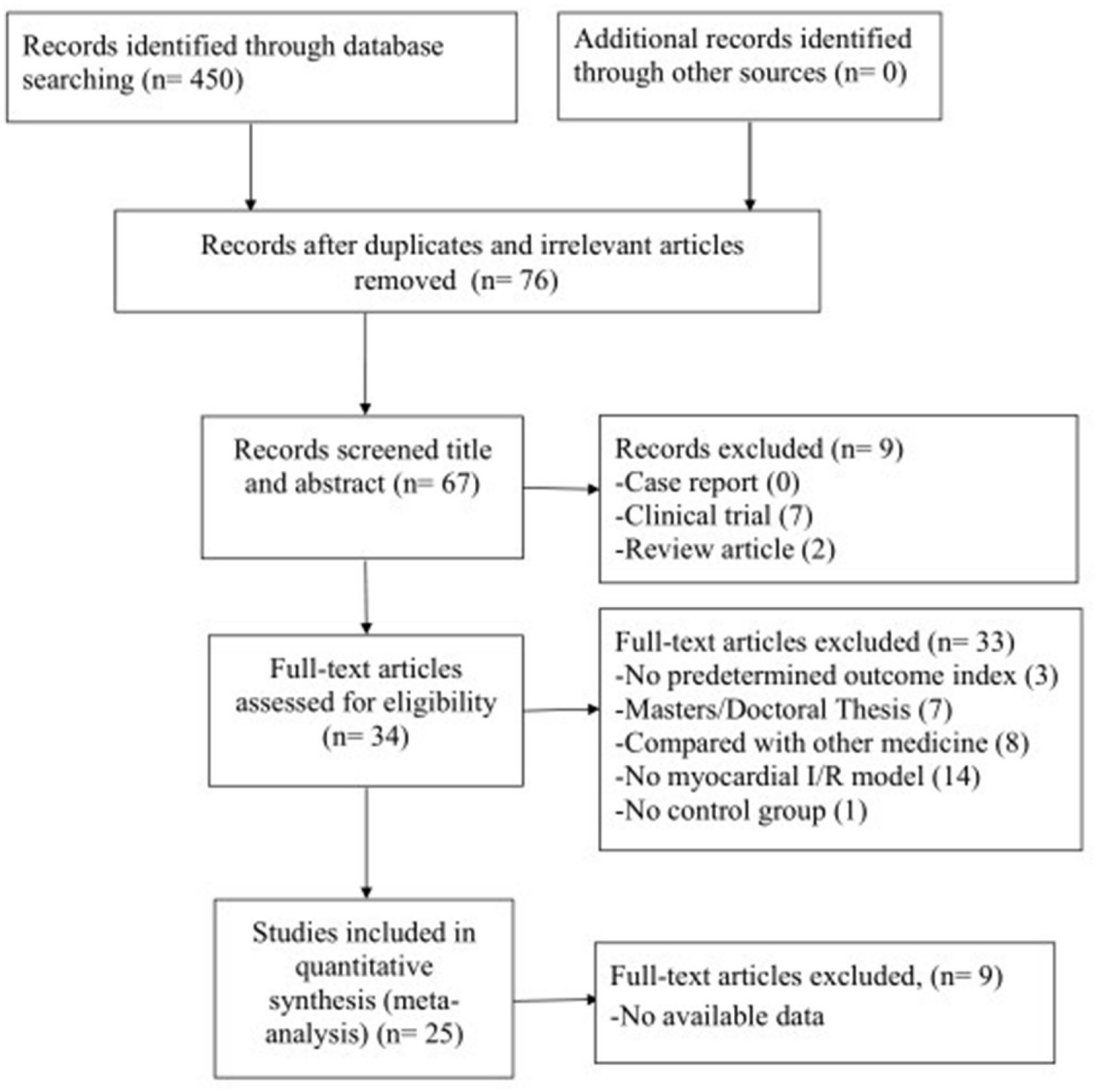
Summary of the process for identifying candidate studies.

We searched 450 potential studies from the database by literature retrieval, of which 374 repetitive and irrelevant studies were excluded. After screening titles and abstracts, 9 studies were excluded because they were case reports, clinical trials or review articles. Reading the remaining full-text articles, 33 articles were excluded if: (1) not predetermined outcome index; (2) not published in peer-review journals; (3) compared with other medicine; (4) no myocardial I/R model; (5) no control group. Then, we removed 9 studies in which data of result is not available. Finally, 25 eligible studies (Xu et al., 1997; Wan et al., 1998; Liang et al., 1999, 2000; Duan et al., 2000; Zhang et al., 2003, 2007, 2015, 2016; Wang et al., 2005; Li et al., 2006; Xu and Zhang, 2006; Chen et al., 2007; Tang et al., 2007; Yang and Rui, 2007; Hu et al., 2008; Shang et al., 2008; Yang et al., 2008; Gu et al., 2009; Li and Li, 2010; Liu and Niu, 2011; Zhai et al., 2011; Lv et al., 2012, 2016; Zhao et al., 2012) were included in qualitative analysis (Figure 2).

### Characteristics of included studies

Twenty-two studies (Xu et al., 1997; Wan et al., 1998; Liang et al., 1999, 2000; Duan et al., 2000; Zhang et al., 2003, 2007; Wang et al., 2005; Li et al., 2006; Xu and Zhang, 2006; Chen et al., 2007; Tang et al., 2007; Yang and Rui, 2007; Hu et al., 2008; Shang et al., 2008; Yang et al., 2008; Gu et al., 2009; Li and Li, 2010; Liu and Niu, 2011; Zhai et al., 2011; Zhao et al., 2012; Lv et al., 2016) were published in Chinese, and 3 studies (Lv et al., 2012; Zhang et al., 2015, 2016) were published in English between 1997 and 2016. Male/female Sprague Dawley rats were used in 11 studies (Duan et al., 2000; Li et al., 2006; Chen et al., 2007; Yang et al., 2008; Gu et al., 2009; Li and Li, 2010; Liu and Niu, 2011; Lv et al., 2012, 2016; Zhao et al., 2012; Zhang et al., 2015, 2016), male/female Wistar rats in 7 studies (Wan et al., 1998; Liang et al., 1999; Xu and Zhang, 2006; Zhang et al., 2007; Hu et al., 2008; Shang et al., 2008; Zhai et al., 2011), male/female Kunming mice in 1 study (Li et al., 2006), and male/female rabbits in 6 studies (Xu et al., 1997; Liang et al., 2000; Zhang et al., 2003; Wang et al., 2005; Tang et al., 2007; Yang and Rui, 2007). To induce anesthesia, 2 studies (Zhang et al., 2007; Zhai et al., 2011) used chloral hydrate; 1 study (Zhang et al., 2015) used sevoflurane; 8 studies (Liang et al., 1999, 2000; Zhang et al., 2003, 2007, 2016; Xu and Zhang, 2006; Tang et al., 2007; Li and Li, 2010) used pentobarbital sodium; 1 study (Duan et al., 2000) used babital sodium; 1 study (Yang et al., 2008) used ethyl ether; and 9 studies (Wang et al., 2005; Li et al., 2006; Chen et al., 2007; Yang and Rui, 2007; Hu et al., 2008; Shang et al., 2008; Gu et al., 2009; Liu and Niu, 2011; Zhao et al., 2012) used urethane, while anaesthetic was not mentioned in the other 2 studies (Zhang et al., 2007; Lv et al., 2012). All myocardial I/R models were established by ligation of the LAD. Among the dose use of Lig, 2 studies (Hu et al., 2008; Shang et al., 2008)used 200 mg·kg^−1^;1 study (Li et al., 2006) used 100 mg·kg^−1^; 3 studies (Duan et al., 2000; Gu et al., 2009; Liu and Niu, 2011) used 50 mg·kg^−1^; 2 studies (Liang et al., 1999, 2000) used 40 mg·kg^−1^; 2 studies (Li and Li, 2010; Lv et al., 2012) used 30 mg·kg^−1^; 1 study (Zhang et al., 2016) used 27 mg·kg^−1^; 1 study (Xu and Zhang, 2006) used 25 mg·kg^−1^; 5 studies (Xu et al., 1997; Wan et al., 1998; Zhang et al., 2003; Tang et al., 2007; Zhai et al., 2011) used 20 mg·kg^−1^;1 study (Yang et al., 2008) used 18 mg·kg^−1^;1 study (Zhang et al., 2007) used 15 mg·kg^−1^; 4 studies (Li et al., 2006; Yang and Rui, 2007; Zhang et al., 2015; Lv et al., 2016) used 10 mg·kg^−1^;1 study (Zhao et al., 2012) used 4 mg·kg^−1^;the remaining 1 study (Wang et al., 2005) used 2 mg·kg^−1^.MI size was utilized as outcome measure in 10 studies (Liang et al., 1999, 2000; Wang et al., 2005; Tang et al., 2007; Yang and Rui, 2007; Hu et al., 2008; Li and Li, 2010; Zhai et al., 2011; Zhao et al., 2012; Zhang et al., 2015). Incidence of ventricular fibrillation (VF) and incidence of ventricular tachycardia (VT) was reported in 2 studies (Liang et al., 1999, 2000), level of ST-segment elevation was reported in 1 study (Wang et al., 2005), but LVEF and FS was not mentioned. Lactate dehydrogenase (LDH) was reported in 11 studies (Liang et al., 1999, 2000; Zhang et al., 2003; Tang et al., 2007; Yang and Rui, 2007; Hu et al., 2008; Yang et al., 2008; Zhai et al., 2011; Zhao et al., 2012; Lv et al., 2016), creatine kinase (CK) in 8 studies (Li et al., 2006; Tang et al., 2007; Yang and Rui, 2007; Hu et al., 2008; Yang et al., 2008; Gu et al., 2009; Li and Li, 2010; Zhai et al., 2011), creatine kinase-MB (CK-MB) in 5 studies (Li et al., 2006; Liu and Niu, 2011; Lv et al., 2012, 2016; Zhao et al., 2012; Zhang et al., 2016), aspartate transaminase (AST) in 1 study (Tang et al., 2007), cTnT in 1 study (Liang et al., 1999), cTnI in 1 study (Zhao et al., 2012), myocardial cell apoptotic index in 3 studies (Duan et al., 2000; Chen et al., 2007; Zhang et al., 2007), superoxide dismutase (SOD) in 9 studies (Xu et al., 1997; Wan et al., 1998; Xu and Zhang, 2006; Hu et al., 2008; Shang et al., 2008; Gu et al., 2009; Li and Li, 2010; Liu and Niu, 2011; Lv et al., 2012), and malondialdehyde (MDA) in 9 studies (Xu et al., 1997; Wan et al., 1998; Hu et al., 2008; Shang et al., 2008; Gu et al., 2009; Li and Li, 2010; Lv et al., 2012; Zhao et al., 2012), glutathione (GSH) in 3 studies (Wan et al., 1998; Xu and Zhang, 2006; Hu et al., 2008), phosphothreonine kinase (p-Akt) in 2 studies (Lv et al., 2012, 2016), caspase-3 in 1 study (Zhang et al., 2007), NO in 4 studies (Xu and Zhang, 2006; Yang and Rui, 2007; Gu et al., 2009; Li and Li, 2010), tumor necrosis factor-α (TNF-α) in 1 study (Hu et al., 2008), heat shock protein (HSP) in 1 study (Chen et al., 2007), IL-6 in 2 studies (Hu et al., 2008; Shang et al., 2008), creatine phosphokinase (CPK) in 1 study (Zhang et al., 2003), and ATP in1 study (Lv et al., 2016). The overall characteristics of included studies are shown in Table 1.

**Table 1:** Characteristics of the 25 included studies.

**Study (years)**	**Species (Sex, *n* = experimental /control group)**	**Weight**	**Model (method)**	**Anesthetic**	**Treatment group (method to astragal sides)**	**Control group**	**Outcome index (time)**	**Intergroup differences**
Li and Li, 2010	SD rats (male, 10/10)	180–200 g	Block LAD for 30 min then reflow for 60 min	Pentobarbital sodium (40 mg/kg, 1%)	By intraperitoneal injection of ligustrazine (30 mg/kg) 10 min before establishing model	By intraperitoneal injection of isasteric NS 10 min before establishing model	Myocardial infarct size (IA/LVA) LDH CK MDA SOD NO eNOS	*P* < 0.05 *P* < 0.05 *P* < 0.05 *P* < 0.05 *P* < 0.05 *P* < 0.05 *P* < 0.05
Zhai et al., 2011	Wistar rats (male, 16/16)	250–300 g	Block LAD for 30 min then reflow for 120 min	Chloral hydrate (250 mg/kg, 10%)	By intravenous injection of ligustrazine (20 mg/kg) 20 min before establishing model	By intravenous injection of nothing before establishing model	Myocardial infarct size (IA/LVA) CK LDH Bcl-2 Bax	*P* < 0.05 *P* < 0.01 *P* < 0.01 *P* < 0.05 *P* < 0.05
Zhao et al., 2012	SD rats (male, 12/12)	250–300 g	Block LAD for 30 min then reflow for 120 min	Urethane (50 mg/kg, 20%)	By intravenous injection of ligustrazine (4 mg/kg) 10 min before establishing model	By intravenous injection of nothing before molding 10 min before establishing model	Myocardial infarct size (IA/AR) CK-MB LDH cTnI SOD MDA Bcl-2 Bax	*P* < 0.05 *P* < 0.05 *P* < 0.05 *P* < 0.05 *P* < 0.05 *P* < 0.05 *P* < 0.05 *P* < 0.05
Yang et al., 2008	SD rats (male, 16/10)	210–230 g	Block LAD for 30 min then reflow for 360 min	Ether	By intravenous injection of ligustrazine (18 mg/kg) before establishing model, once a day, for 2 days	By intravenous injection of isasteric NS before establishing model, once a day, for 2 days	LDH CK	*P* < 0.05 *P* < 0.05
Liang et al., 1999	Wistar rats (male, 8/8)	300–400 g	Block LAD for 30 min then reflow for 120 min	Pentobarbital sodium (60 mg/kg)	By intravenous injection of ligustrazine (40 mg/kg) 5 min earlier before reperfusion	By intravenous injection of nothing 5 min earlier before reperfusion	Myocardial infarct size (IA/LVA) LDH Incidence of VF Incidence of VT	*P* < 0.05 *P* < 0.05 *P* < 0.05 *P* < 0.05
Zhang et al., 2007	Wistar rats (male/female, 10/10)	250-260 g	Block LAD for 1 h then reflow for 24 h	Not mentioned	By intraperitoneal injection of ligustrazine (15 mg/kg) 60 min earlier before establishing model	By intraperitoneal injection of nothing 60 min earlier before establishing model	Apoptotic index Caspase-3 Fas Caspase-8	*P* < 0.01 *P* < 0.01 *P* < 0.01 *P* < 0.01
Xu and Zhang, 2006	Wistar rats (male/female, 15/15)	218–262 g	Block LAD for 30 min then reflow for 60 min	Pentobarbital sodium (45 mg/kg)	By intravenous injection of ligustrazine (25 mg/kg) 10 min before establishing model	By intravenous injection of isasteric NS before establishing model	SOD GSH NO	*P* < 0.01 *P* < 0.01 *P* < 0.01
Wan et al., 1998	Wistar rats (male, 10/10)	200–250 g	Block LAD for 30 min then reflow for 20 min	pentobarbital sodium (50 mg/kg)	By intraperitoneal injection of ligustrazine (20 mg/kg) 60 min earlier before establishing model	By intraperitoneal injection of nothing 60 min earlier before establishing model	MDA SOD GSH	*P* < 0.01 *P* < 0.01 *P* < 0.01
Liu and Niu, 2011	SD rats (male, 12/12)	222–242 g	Block LAD for 45 min then reflow for 60 min	Urethane (1 mg/kg, 20%)	By intravenous injection of ligustrazine (50 mg/kg) before reperfusion	By intravenous injection of nothing (50 mg/kg) before reperfusion	SOD MDA Bax Bcl-2	*P* < 0.01 *P* < 0.01 *P* < 0.01 *P* < 0.01
Duan et al., 2000	SD rats (female, 18/18)	185–215 g	Block LAD for 10 min than reflow for 120 min	Barbital sodium (50 mg/kg)	By intravenous injection of ligustrazine (50 mg/kg) before reperfusion	By intravenous injection of isasteric NS before reperfusion	Apoptotic index	*P* < 0.01
Shang et al., 2008	Wistar rats (male/female, 10/10)	200–300 g	Block LAD for 30 min then reflow for 120 min	Urethane (5 ml/kg)	By intravenous injection of ligustrazine (200 mg/kg), for 10 days, before establishing model	By intravenous injection of isasteric NS, for 10 days, before establishing model	SOD MDA TNF-α IL-6 HSP25 p28MAPK	*P* < 0.01 *P* < 0.01 *P* < 0.01 *P* < 0.01 *P* < 0.01 *P* < 0.01
Gu et al., 2009	SD rats (male, 10/10)	250–300 g	Block LAD for 30 min then reflow for 180 min	Urethane (5 ml/kg)	By intravenous injection of ligustrazine (50 mg/kg, dilution to 2 ml) 2 min earlier before reperfusion	By intravenous injection of isasteric NS earlier 2 min before reperfusion	CK SOD MDA NO eNOS	*P* < 0.01 *P* < 0.01 *P* < 0.05 *P* < 0.05 *P* < 0.01
Hu et al., 2008	Wistar rats (male/female, 20/20)	200–300 g	Block LAD for 30 min then reflow for 120 min	Urethane (5 ml/kg)	By intragastric gavage of ligustrazine (200 mg/kg) before establishing model, once a day, for 10 days	By intragastric gavage of isasteric NS before establishing model, once a day, for 10 days	Myocardial infarct size (IA/LVA) SOD GSH MDA LDH CK TNF-α IL-6 ST-segmentelevation	*P* < 0.05 *P* < 0.01 *P* < 0.01 *P* < 0.01 *P* < 0.01 *P* < 0.01 *P* < 0.01 *P* < 0.01 *P* < 0.01
Li et al., 2006	Kunming mice (male/female, 8/8)	20–30 g	Byintraperitoneal injection of pituitrin (30 u/kg), 30 min later, intraperitoneal injection of pituitrin glyceryltrinitrate (10 mg/kg)	Urethane (2 g/kg, 20%)	By intragastric gavage of ligustrazine (25 ml/kg, 40%), once a day, for 7 days	By intragastric gavage of isasteric NS, once a day, for 7 days	Myocardial infarct size CK CK-MB LDH cTnT	*P* < 0.01 *P* < 0.01 *P* < 0.01 *P* < 0.01 *P* < 0.01
Chen et al., 2007	SD rats (male/female, 8/8)	65–75 g	Block LAD for 30 min then reflow for 120 min	Urethane	By intraperitoneal injection of ligustrazine (100 mg/kg) 48 h before establishing model	By intraperitoneal injection of isastericNS 48 h before establishing model	Apoptosis indexs HSP	*P* < 0.05 *P* < 0.05
Lv et al., 2016	SD rats (male, 8/8)	260–300 g	Block LAD for 35 min then reflow for 120 min	Chloral hydrate (10%)	By intravenous injection of ligustrazine (10 mg/kg) 5 min earlier before establishing model	By intravenous injection of nothing before establishing model	CK-MB LDH ATP p-Akt	*P* < 0.05 *P* < 0.05 *P* < 0.05 *P* < 0.05
Zhang et al., 2015	SD rats (male, 10/10)	250–300 g	Block LAD	Sevoflurane	By intravenous injection of ligustrazine (10 mg/kg)	By intravenous injection of nothing	Myocardial infarct size (IA/AR)	*P* < 0.01
Lv et al., 2012	SD rats (male, 10/10)	250–280 g	Block LAD for 25 min then reflow for 120 min	Not mentioned	By intravenous injection of ligustrazine (30 mg/kg) 5 min before establishing model	By intravenous injection of nothing 5 min before establishing model	CK-MB MDA SOD p-Akt eNOS	*P* < 0.05 *P* < 0.05 *P* < 0.05 *P* < 0.05 *P* < 0.05
Zhang et al., 2016	SD rats (male, 15/15)	300–350 g	Left ventricle injection of sodium laurate (2 g/L, 0.2 mL)	Pentobarbital sodium (40 mg/kg)	By intravenous injection of ligustrazine (27 mg/kg) once a day, for 2 weeks, before establishing model	By intravenous injection of nothing, once a day, for 2 weeks, before establishing model	CK-MB LVSP LVEDP +dp/dtmax –dp/dtmax HR	*P* < 0.05 *P* < 0.05 *P* < 0.05 *P* < 0.05 *P* < 0.05 *P* < 0.05
Yang and Rui, 2007	New Zealand white rabbits (male/female, 8/8)	2.0–2.5 kg	Block LAD for 40 min then reflow for 120 min	Urethane (2 ml/kg, 20%)	By intravenous injection of ligustrazine (10 mg/kg) before establishing model	By Intravenousinjection of isasteric NS before establishing model	Myocardial infarct size (IA/LVA) LDH CK NO	*P* < 0.01 *P* < 0.01 *P* < 0.01 *P* < 0.01
Xu et al., 1997	Rabbits (male/female, 10/10)	2.0–2.8 kg	Block LAD for 40 min then reflow for 20 min	Polyurethane (1.0 g/kg)	By intravenous injection of ligustrazine (20 mg/kg) 20 min before establishing model	By intravenous injection of nothing before establishing model	LVSP +dp/dtmax –dp/dtmax MDA SOD GSH-PX	*P* < 0.05 *P* < 0.01 *P* < 0.01 *P* < 0.05 *P* < 0.05 *P* < 0.05
Tang et al., 2007	New Zealand white rabbits (male, 8/8)	2.1–3.2 kg	Block LAD for 30 min then reflow for 120 min	Pentobarbital sodium (30 mg/kg)	By intraperitoneal injection of ligustrazine (20 mg/kg) 60 min before establishing model	By intraperitoneal injection of nothing 60 min before establishing model	Myocardial infarct size (IA/AR) LVSP LVEDP +dp/dtmax –dp/dtmax CK LDH AST	*P* < 0.01 *P* < 0.01 *P* < 0.01 *P* < 0.01 *P* < 0.01 *P* < 0.01 *P* < 0.01 *P* < 0.01
Wang et al., 2005	New Zealand white rabbits (male/female, 15/15)	2.8–3.3 kg	Block LAD for 90 min then reflow for 120 min	Urethane (1 g/kg, 20%)	By intravenous injection of ligustrazine (2 mg/kg) before establishing model	Intravenous isasteric NS before establishing model	Myocardial infarct size (IA/LVA) ST-segmentelevation	*P* < 0.01 *P* < 0.01
Liang et al., 2000	Rabbits (male, 6/6)	2.1–3.5 kg	Block LAD for 30 min then reflow for 120 min	Pentobarbital sodium (30 mg/kg)	By intravenous injection of ligustrazine (40 mg/kg) before reperfusion	By intravenous injection of nothing before reperfusion	Myocardial infarct size (IA/AR) LDH Incidence of VF Incidence of VT	*P* < 0.05 *P* < 0.05 *P* < 0.05 *P* < 0.05
Zhang et al., 2003	Rabbits (male, 8/8)	Not mentioned	Block LAD for 45 min than reflow for 180 min	Pentobarbital sodium (30 mg/kg, 3%)	By intravenous injection of ligustrazine (20 mg/kg) before reperfusion	By intravenous injection of nothing before reperfusion	LDH CPK	*P* < 0.01 *P* < 0.01

*SD rats, Sprague-Dawley; LAD, the left anterior descending coronary artery; Min, minutes; NS, normal saline; IA/LVA, infarct area/left ventricular area; IA/AR, infarct area/area at risk; SOD, superoxide dismutase; MDA, malondialdehyde; eNOS, endothelial nitric oxide synthase; CK, creatine kinase; LDH, lactate dehydrogenase; CK-MB, creatine kinase-MB; cTnT, cardiac troponin T; cTnI, cardiac troponin I; VF, ventricular fibrillation; VT, ventricular tachycardia; GSH, glutathione synthetase; TNF-α, tumor necrosis factor-α; HR, heart rate; HSP, heat shock protein; AST, aspartate transaminase; LVSP, left ventricular systolic pressure; LVEDP, left ventricular end-diastolic pressure; CPK, creatine phosphokinase*.

### Study quality

The quality score of studies ranged from 2 to 6. All studies were publications in a peer reviewed journal. Twelve studies (Wang et al., 2005; Li et al., 2006; Tang et al., 2007; Zhang et al., 2007, 2015, 2016; Hu et al., 2008; Shang et al., 2008; Gu et al., 2009; Lv et al., 2012, 2016; Zhao et al., 2012) reported control of temperature. Twenty-one studies (Wan et al., 1998; Liang et al., 1999, 2000; Duan et al., 2000; Zhang et al., 2003, 2007; Wang et al., 2005; Li et al., 2006; Xu and Zhang, 2006; Chen et al., 2007; Tang et al., 2007; Yang and Rui, 2007; Hu et al., 2008; Shang et al., 2008; Yang et al., 2008; Gu et al., 2009; Li and Li, 2010; Liu and Niu, 2011; Zhai et al., 2011; Zhao et al., 2012; Lv et al., 2016) described random allocation to treatment or control. One study (Zhang et al., 2015) mentioned blinded induction of model. None of studies described blinded assessment of outcome. Twenty-four studies (Xu et al., 1997; Wan et al., 1998; Liang et al., 1999, 2000; Duan et al., 2000; Zhang et al., 2003, 2007, 2015, 2016; Wang et al., 2005; Li et al., 2006; Xu and Zhang, 2006; Chen et al., 2007; Tang et al., 2007; Yang and Rui, 2007; Hu et al., 2008; Shang et al., 2008; Yang et al., 2008; Gu et al., 2009; Li and Li, 2010; Liu and Niu, 2011; Zhai et al., 2011; Zhao et al., 2012; Lv et al., 2016) used anesthetic without significant intrinsic vascular protection activity. No study describes appropriate animal model (aged, diabetic, or hypertensive) and the sample size calculation. Two studies (Zhang et al., 2015, 2016) stated compliance with animal welfare regulations and 1 study (Zhang et al., 2015) declared statement of potential conflict of interests. The methodological quality is concluded in Table 2.

**Table 2:** Risk of bias of the included studies.

**Study**	**A**	**B**	**C**	**D**	**E**	**F**	**G**	**H**	**I**	**J**	**Total**
Li and Li, 2010	√		√			√					3
Zhai et al., 2011	√		√			√					3
Zhao et al., 2012	√	√	√			√					4
Yang et al., 2008	√		√			√					3
Liang et al., 1999	√		√			√					3
Zhang et al., 2007	√	√	√			√					4
Xu and Zhang, 2006	√		√			√					3
Wan et al., 1998	√		√			√					3
Liu and Niu, 2011	√		√			√					3
Duan et al., 2000	√		√			√					3
Shang et al., 2008	√	√	√			√					4
Gu et al., 2009	√	√	√			√					4
Hu et al., 2008	√	√	√			√					4
Li et al., 2006	√	√	√			√					4
Chen et al., 2007	√		√			√					3
Lv et al., 2016	√	√	√			√					4
Zhang et al., 2015	√	√		√		√			√	√	6
Lv et al., 2012	√	√									2
Zhang et al., 2016	√	√				√			√		4
Yang and Rui, 2007	√		√			√					3
Xu et al., 1997	√					√					2
Tang et al., 2007	√	√	√			√					4
Wang et al., 2005	√	√	√			√					4
Liang et al., 2000	√		√			√					3
Zhang et al., 2003	√		√			√					3

*Studies fulfilling the criteria of: A, peer reviewed publication; B, control of temperature; C, random allocation to treatment or control; D, blinded induction of model; E, blinded assessment of outcome; F, use of anesthetic without significant intrinsic vascular protection activity; G, appropriate animal model (aged, diabetic, or hypertensive); H, sample size calculation; I, compliance with animal welfare regulations; J, statement of potential conflict of interests*.

### Effectiveness

#### MI size

**Figure 3:**
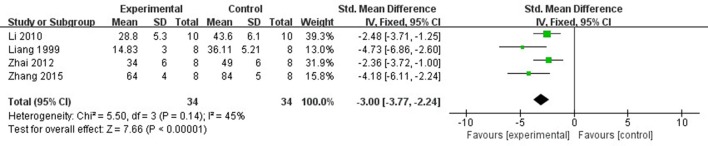
The forest plot: effects of ligustrazine for decreasing the myocardial infarction size (infarct area/left ventricular area) in rats compared with control group.

**Figure 4:**
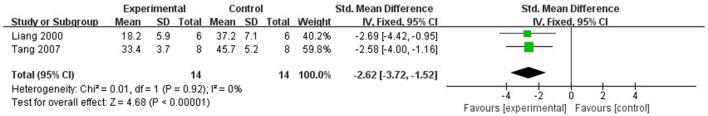
The forest plot: effects of ligustrazine for decreasing the myocardial infarction size (infarct area/area at risk) in rabbits compared with control group.

Ten studies (Liang et al., 1999, 2000; Wang et al., 2005; Tang et al., 2007; Yang and Rui, 2007; Hu et al., 2008; Li and Li, 2010; Zhai et al., 2011; Zhao et al., 2012; Zhang et al., 2015) utilized MI size as outcome measure. All of them showed significant effect of Lig for decreasing the MI size (*P* < 0.05). Because the methods to calculate MI size are different, we divided the studies into two parts as follow: (1) Infarct area/left ventricular area (IA/LVA): Five studies (Liang et al., 1999; Hu et al., 2008; Li and Li, 2010; Zhai et al., 2011; Zhang et al., 2015) were carried out in rats and 2 studies (Wang et al., 2005; Yang and Rui, 2007) were carried out in rabbits. Four studies (Liang et al., 1999; Li and Li, 2010; Zhai et al., 2011; Zhang et al., 2015) used male rats, whereas 1 study (Hu et al., 2008) used male/female rats. Meta-analysis of above 4 studies (Liang et al., 1999; Li and Li, 2010; Zhai et al., 2011; Zhang et al., 2015) showed significant effect of Lig for decreasing the MI size compared with control group (*n* = 34, SMD −3.00, 95% CI [2.24 to −3.77], *P* < 0.01; heterogeneity: χ^2^ = 5.50, *df* = 3 (*P* = 0.14); *I*^2^ = 45%) (Figure 3). They failed to pool meta-analysis of 2 studies in rabbits, because the time of MI model induced was by blocking LAD for 40 min (Yang and Rui, 2007) vs. 90 min (Wang et al., 2005). (2) Infarct area/area at risk (IA/AR): Two studies (Liang et al., 2000; Tang et al., 2007) used in rabbits and one study (Zhao et al., 2012) used in rats. Meta-analysis of 2 studies in rabbits (Liang et al., 2000; Tang et al., 2007) showed significant effect of Lig for decreasing the MI size compared with control group (*n* = 14, SMD −2.62, 95% CI [1.52 to −3.72], *P* < 0.01; heterogeneity: χ^2^ = 0.01, *df* = 1 (*P* = 0.92); *I*^2^ = 0%; Figure 4).

#### Cardiac enzymes and/or troponin

**Figure 5:**
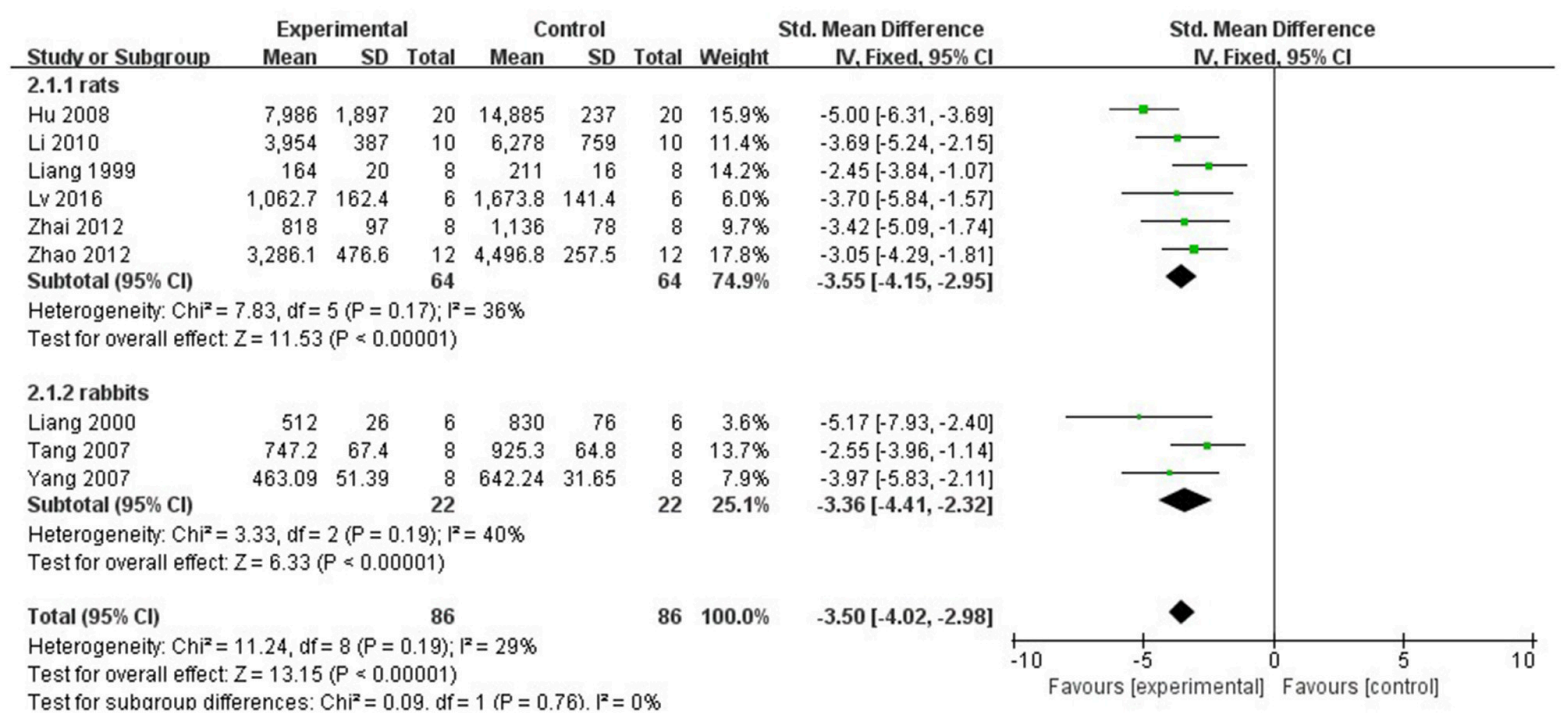
The forest plot: effects of ligustrazine for decreasing lactate dehydrogenase compared with control group.

**Figure 6:**
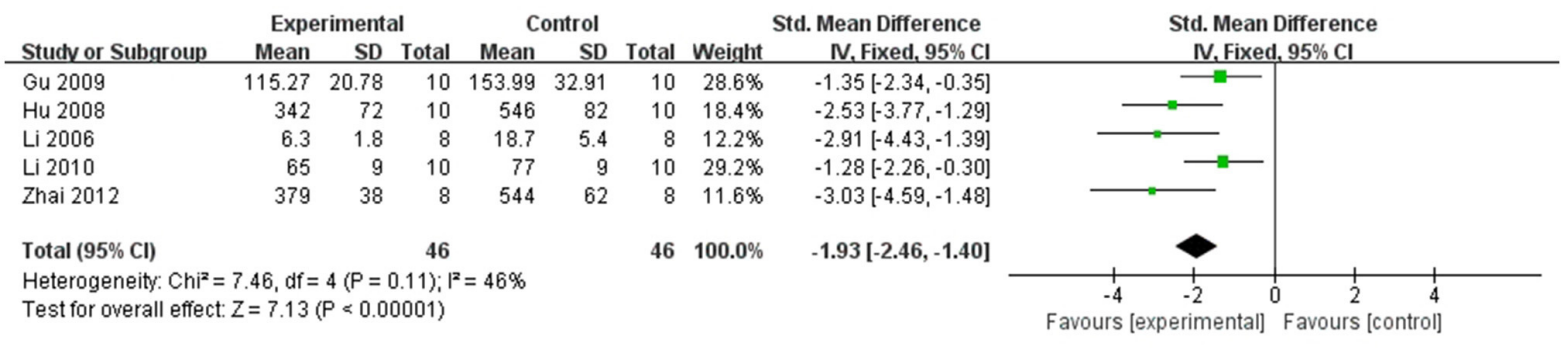
The forest plot: effects of ligustrazine for decreasing creatine kinase in rats compared with control group.

**Figure 7:**
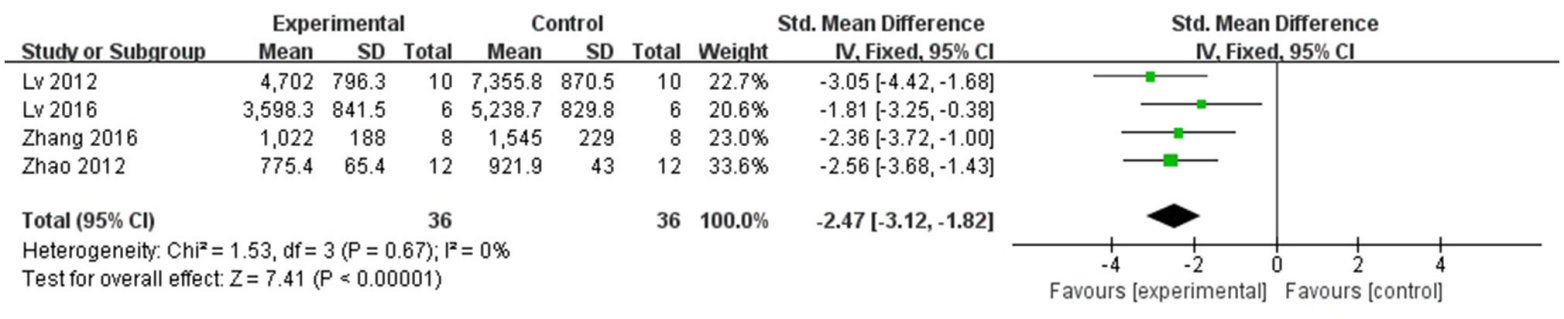
The forest plot: effects of ligustrazine for decreasing creatine kinase-MB in rats compared with control group.

In rat studies, 7 studies used LDH as the outcome (Liang et al., 1999; Hu et al., 2008; Yang et al., 2008; Li and Li, 2010; Zhai et al., 2011; Zhao et al., 2012; Lv et al., 2016). Meta-analysis of 6 studies (Liang et al., 1999; Hu et al., 2008; Li and Li, 2010; Zhai et al., 2011; Zhao et al., 2012; Lv et al., 2016) showed significant effect of Lig for decreasing the LDH compared with control group (*n* = 64, SMD −3.55, 95% CI [−2.95 to −4.15], *P* < 0.01; heterogeneity: χ^2^ = 7.83, df = 5 (*P* = 0.17); *I*^2^ = 36%) (Figure 5). We removed 1 study (Yang et al., 2008) that used LDH as the outcome, in which time of reperfusion was 360 min vs. 90 min in above studies. Meta-analysis of 5 studies (Li et al., 2006; Hu et al., 2008; Gu et al., 2009; Li and Li, 2010; Zhai et al., 2011) in rats showed significant effect of Lig for decreasing the CK compared with control group (*n* = 46, SMD −1.93, 95% CI [1.40 to −2.46], *P* < 0.01; heterogeneity: χ^2^ = 7.46, *df* = 4 (*P* = 0.11); *I*^2^ = 46%; Figure 6). Meta-analysis of 4 studies in rats (Lv et al., 2012, 2016; Zhao et al., 2012; Zhang et al., 2016) showed significant effect of Lig for decreasing the CK-MB compared with control group (*n* = 36, SMD −2.47, 95% CI [1.82 to −3.12], *P* < 0.01; heterogeneity: χ^2^ = 1.53, *df* = 3 (*P* = 0.67); *I*^2^ = 0%) (Figure 7). Lig significantly decreased AST (Tang et al., 2007), cTnT (Liang et al., 1999) or cTnI (Zhao et al., 2012) in rats respectively compared with control (*P* < 0.05).

In rabbit studies, meta-analysis of 3 studies (Liang et al., 2000; Tang et al., 2007; Yang and Rui, 2007) showed significant effect of Lig for decreasing the LDH compared with control group (*n* = 22, SMD −3.36, 95% CI [2.32 to −4.41], *P* < 0.00001; heterogeneity: χ^2^ = 3.33, *df* = 2 (*P* = 0.19); *I*^2^ = 40%) (Figure 5). Two studies (Tang et al., 2007; Yang and Rui, 2007) in rabbits showed significant effect of Lig for decreasing the CK compared with control group (*P* < 0.05). They failed to pool meta-analysis because the time of MI model induced was by blocking LAD for 40 min (Yang and Rui, 2007). One study (Wang et al., 2005) reported that Lig can improve the ST-segment depression compared with control (*P* < 0.05). There was no study involving LVEF and FS as outcome measure.

#### Cardioprotective mechanisms

**Figure 8:**
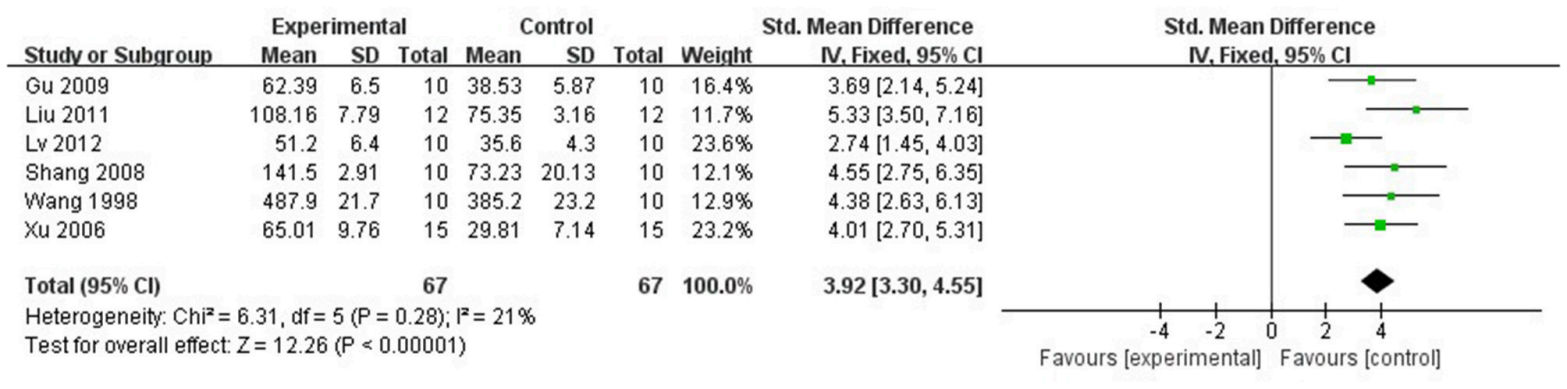
The forest plot: effects of ligustrazine for increasing superoxide dismutase compared with control group.

**Figure 9:**
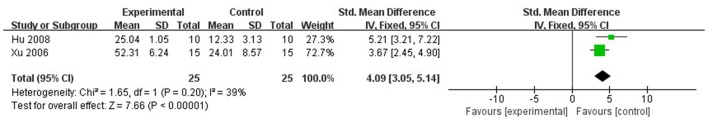
The forest plot: effects of ligustrazine for increasing glutathione compared with control group.

Compared with controls, meta-analysis of 6 studies (Wan et al., 1998; Xu and Zhang, 2006; Shang et al., 2008; Gu et al., 2009; Liu and Niu, 2011; Lv et al., 2012) showed that Lig significantly increased SOD (*n* = 67, MD 3.92, 95% CI [3.30–4.55], *P* < 0.01; heterogeneity: χ^2^ = 6.31, *df* = 5 (*P* = 0.28); *I*^2^ = 21%; Figure 8); 2 studies (Liang et al., 2000; Lv et al., 2012) for increasing GSH (*n* = 25, SMD 4.09, 95% CI [3.05–5.14], *P* < 0.01; heterogeneity: χ^2^ = 1.65, *df* = 1 (*P* = 0.20); *I*^2^ = 39%; Figure 9); 9 studies (Xu et al., 1997; Wan et al., 1998; Hu et al., 2008; Shang et al., 2008; Gu et al., 2009; Li and Li, 2010; Liu and Niu, 2011; Lv et al., 2012; Zhao et al., 2012) for reducing MDA (*P* < 0.05); 4 studies (Xu and Zhang, 2006; Yang and Rui, 2007; Gu et al., 2009; Li and Li, 2010) for increasing NO (*P* < 0.05); 1 study (Zhang et al., 2003) for increasing CPK (*P* < 0.05); 3 studies (Duan et al., 2000; Chen et al., 2007; Zhang et al., 2007) for decreasing apoptotic index (*P* < 0.05); 2 studies (Lv et al., 2012, 2016) for increasing phosphothreonine kinase (p-Akt) (*P* < 0.05); 1 study (Zhang et al., 2007) for decreasing caspase-3 (*P* < 0.05); 1 study (Hu et al., 2008) for decreasing TNF-α (*P* < 0.05); 1 study (Chen et al., 2007) for decreasing HSP (*P* < 0.05); 2 studies (Hu et al., 2008; Shang et al., 2008) for decreasing IL-6 (*P* < 0.05); 1 study (Lv et al., 2016) for increasing ATP (*P* < 0.05).

## Discussion

### Summary of evidence

Twenty-five studies involving 556 animals were identified. The findings from present study demonstrated that Lig exerts cardioprotection inmyocardial I/R injury, largely through antioxidant, anti-inflammatory, anti-apoptotic activities, and improving coronary blood flow and myocardial metabolism via multiple signaling pathways. Despite significant positive results, we should treat the results consciously because of the flaw of methodological quality.

### Limitations

Some limitations should be considered while interpreting this study. First, we only included studies from Chinese and English databases. The absence of studies written in other languages may lead to certain degree of selective bias (Nolting et al., 2012). Second, study quality was considered as moderate, which ranged from 2 to 6 points, may affecting the accuracy of the results (Landis et al., 2012). Third, none of the included studies used animals with relevant comorbidities, which are not in conformity with pathophysiology in patients with MI (Landis et al., 2012). Finally, although the heart protective effect of estrogen has been reported both in clinical and preclinical studies (Menazza et al., 2017), 10 including studies (Duan et al., 2000; Wang et al., 2005; Li et al., 2006; Xu and Zhang, 2006; Chen et al., 2007; Tang et al., 2007; Yang and Rui, 2007; Zhang et al., 2007; Hu et al., 2008; Shang et al., 2008)adopted female animals in this study.

### Implications

Poor design of animal research is considered as a roadblock to translate animal research into promising preclinical drug treatments for human disease (Baginskaite, 2012). In the present study, many domains had flaws in aspects of randomization, allocation concealment, and blinding and sample size calculation, which are the core standards of study design (Moher et al., 2015). A lower-quality study trends toward better outcomes, leading to the global estimated effect overstated (García et al., 2012). Thus, in the future study, we recommended that the experimental research of Lig for MI need be promoted by means of incorporating the ARRIVE guidelines (Kilkenny et al., 2012).

**Figure 10:**
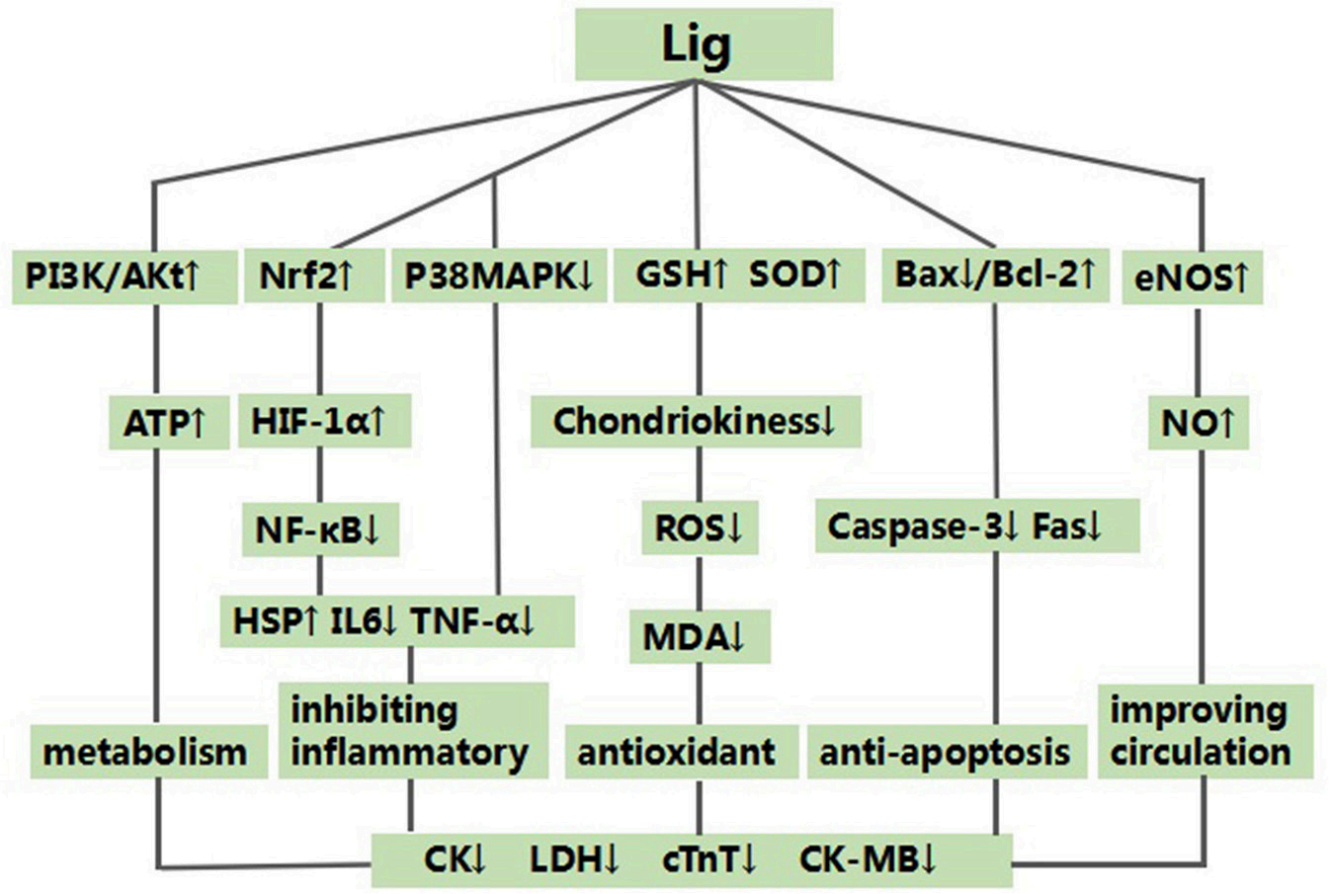
A schematic representation of cardioprotective mechanisms of Ligustrazine for myocardial ischemia/reperfusion injury. ↑ means enhance the expression of relevant protein or pathway. ↓ means inhibit the expression of relevant protein or pathway.

The possible mechanisms of Lig for cardioprotective function are summarized as follows: (1) antioxidant through increasing glutathione (GSH) (Wan et al., 1998; Xu and Zhang, 2006; Hu et al., 2008), and enhancing SOD-induced (Xu et al., 1997; Wan et al., 1998; Xu and Zhang, 2006; Hu et al., 2008; Shang et al., 2008; Gu et al., 2009; Li and Li, 2010; Liu and Niu, 2011; Lv et al., 2012) antioxidant via attenuating chondriokinesis to reduce the release of malondialdehyde (MDA) (Xu et al., 1997; Wan et al., 1998; Xu and Zhang, 2006; Hu et al., 2008; Shang et al., 2008; Gu et al., 2009; Li and Li, 2010; Liu and Niu, 2011; Lv et al., 2012; Zhao et al., 2012); (2) the main anti-inflammatory mechanisms: Lig can inhibit P38MAPK pathway (Qian et al., 2014) and enhance nuclear factor (erythroid-derived 2)-like 2 (Nrf2)/hypoxia-inducible factor 1-alpha (HIF-1α) pathway, and they further inhibited the expression of NF-κB (Lu et al., 2017). Simultaneously, Lig can inhibit the expression of the endothelial cell adhesion molecule of E-selectin, P-selectin and intercellular adhesion molecule-1 (Yang et al., 2008). Ultimately, they can alleviate inflammatory of myocardial I/R injury through inhibiting the expression of tumor necrosis factor-α (Hu et al., 2008), IL- 6 (Hu et al., 2008; Shang et al., 2008; Wang et al., 2017), and/or promoting the expression of HSP (Chen et al., 2007; Shang et al., 2008); (3) inhibition of apoptosis (Duan et al., 2000; Chen et al., 2007; Zhang et al., 2007) through increasing the expression of Bcl-2 (Liu and Niu, 2011; Zhai et al., 2011; Zhao et al., 2012), reducing the expression of Bax protein (Liu and Niu, 2011; Zhai et al., 2011; Zhao et al., 2012) in the myocardium, and down-regulating the expression of caspase and Fas (Zhang et al., 2007); (4) metabolism mechanism through increasing the expression of the protein of p-Akt (Lv et al., 2012, 2016) and content of ATP in myocardium (Lv et al., 2016) with activating PI3K/Akt signal pathway (Lv et al., 2012, 2016); (5) improvement of the circulation by enhancing the expression of NO (Wan et al., 1998; Yang and Rui, 2007; Gu et al., 2009; Li and Li, 2010) via up-regulating the expression of NOS (Li et al., 2006; Xu and Zhang, 2006; Yang and Rui, 2007; Gu et al., 2009). A schematic representation of cardioprotective mechanism of Lig for myocardial I/R injury was summarized in Figure 10. Thus, Ligexerts cardioprotection inmyocardial I/R injury through multiple signaling pathways. Further studies should clarify the exact mechanisms of Lig for MI.

## Conclusion

Our findings indicate that Ligexerted cardioprotective function for myocardial I/R injury largely through antioxidant, anti-inflammatory, anti-apoptosis activities and improving coronary blood flow and myocardial metabolism.

## Author contributions

QZ, YH, and PZ contributed equally to this work. QZ, YH, PZ, QT, XB, YW and GZ designed the study; QZ, YH and PZ collected the data; QZ and YH performed all analyses; All authors contributed to writing of this manuscript.

### Conflict of interest statement

The authors declare that the research was conducted in the absence of any commercial or financial relationships that could be construed as a potential conflict of interest.
